# Future workspace needs flexibility and diversity: A machine learning-driven behavioural analysis of co-working space

**DOI:** 10.1371/journal.pone.0292370

**Published:** 2023-10-18

**Authors:** Jiayu Pan, Tze Yeung Cho, Maoran Sun, Ramit Debnath, Nathan Lonsdale, Chris Wilcox, Ronita Bardhan

**Affiliations:** 1 Sustainable Design Group, Department of Architecture, University of Cambridge, Cambridge, United Kingdom; 2 Department of Engineering, University of Cambridge, Cambridge, United Kingdom; 3 Cambridge Zero and Computer Laboratory, University of Cambridge, Cambridge, United Kingdom; 4 Collective Intelligence and Design Group, Department of Architecture, University of Cambridge, Cambridge, United Kingdom; 5 Division of Humanities and Social Science, California Institute of Technology, Pasadena, CA, United States of America; 6 spacelab_, London, United Kingdom; 7 sense_, London, United Kingdom; TU Wien: Technische Universitat Wien, AUSTRIA

## Abstract

The future of workspace is significantly shaped by the advancements in technologies, changes in work patterns and workers’ desire for an improved well-being. Co-working space is an alternative workspace solution, for cost-effectiveness, the opportunity for diverse and flexible design and multi-use. This study examined the human-centric design choices using spatial and temporal variation of occupancy levels and user behaviour in a flexible co-working space in London. Through a machine-learning-driven analysis, we investigated the time-dependent patterns, decompose space usage, calculate seat utilisation and identify spatial hotspots. The analysis incorporated a large dataset of sensor-detected occupancy data spanning 477 days, comprising more than 140 million (145×10^6^) data points. Additionally, on-site observations of activities were recorded for 13 days spanning over a year, with 110 time instances including more than 1000 snapshots of occupants’ activities, indoor environment, working behaviour and preferences. Results showed that the shared working areas positioned near windows or in more open, connected and visible locations are significantly preferred and utilised for communication and working, and semi-enclosed space on the side with less visibility and higher privacy are preferred for focused working. The flexibility of multi-use opportunity was the most preferred feature for hybrid working. The findings offer data-driven insights for human-centric space planning and design of office spaces in the future, particularly in the context of hybrid working setups, hot-desking and co-working systems.

## 1 Introduction

The modern workspace has undergone significant transformations driven by advancements in information and communication technology and changes in working patterns and human preferences. The recent global pandemic has further accelerated these changes, reshaping daily routines and work practice. Multiple surveys have suggested employees’ desire for keeping work from home as an option, a more flexible work schedule and location and a rising focus on health and well-being [1, 2]. Subsequently, there is a growing interest in local co-working space as an alternative to traditional office setups like open-plan and cubicles to facilitate the new demand [3–5]. These co-working spaces offer an environment where diverse professionals can network and collaborate, providing flexibility, diversity and dynamic work environments at a reduced cost [6–9]. As the demand for flexible working arrangements continues to grow, understanding the spatial and temporal variation of occupancy levels and user activities in co-working spaces becomes crucial to improve the well-being of the occupants.

The availability of enhanced data on spatial qualities and behavioural characteristics provides an opportunity to uncover general patterns for space usage and building system management [10]. Collecting data-driven evidence about spaces, cultures, behaviours and space usage patterns helps form better design and facility management decisions [11, 12]. With the improvement in the smart building and sensor technology, the concept of data-driven and evidence-based design holds immense potential for shaping the future of office design in response to evolving work dynamics and accommodating hybrid working. For instance, consider a scenario where some employees work remotely and only visit the office sporadically, resulting in numerous unoccupied desks. By accurately determining the number of individuals present during work hours through occupancy data, it becomes possible to identify the optimal number of desks needed and adjust the layout for different uses (such as meeting, gathering and working) accordingly. This information allows for a more efficient allocation of spaces based on the actual number of employees, ensuring a dynamic use of space [13]. Additionally, the use of smart sensors could assist the efficient operation of the building energy and lighting system, particularly when the space is vacant [12, 14].

This study presents an exploratory analysis of the occupancy level and occupants’ activities in a co-working office in London after the pandemic. It combines the sensor-detected occupancy data and observed occupants’ activities data and applies the spatial-temporal analysis methods to the dataset. The study fills the knowledge gap of translating the empirical evidence to design insights for the co-working space in the hybrid working setup. The aims of the study can be specified as: 1) capturing temporal variations in occupancy patterns; 2) investigating the distribution of occupancy across zones and calculating seat utilisation rates; 3) identifying spatial hotspots for occupancy and activities; and 4) informing basic space design and planning decisions based on the empirical evidence derived from the data.

The remainder of this paper is structured as follows: Section 2 provides a literature review on studies related to workspace occupancy analysis and activities in co-working spaces, which sets up the background of this study. Section 3 outlines the methods used, including site information, data collection procedures and spatial-temporal analysis and unsupervised learning techniques like clustering analysis. Section 4 presents the results of the analysis, while Section 5 offers a discussion of the findings. Finally, Section 6 concludes the paper by illustrating the study’s limitations and suggesting avenues for future research.

## 2 Literature review

### 2.1 Analysis of workspace occupancy and activity

The analysis and prediction of indoor environment occupancy have been extensively studied due to their significance in understanding occupancy patterns, improving occupants’ comfort and optimizing energy utilization [15–19]. Accurate detection of occupancy in office space is crucial for space utilisation and efficient control of lighting, heating, cooling and ventilating systems to lower the environmental footprint [13]. However, collecting occupancy data has posed challenges due to privacy concerns and the limited infrastructure for precise people sensing within buildings [15]. Moreover, some carryover effects are observed while using the sensors, especially when occupants are aware of the presence of sensors [20]. These challenges are gradually being addressed through the implementation of smart building technologies and sensor systems.

Occupancy measurements can involve the identification of the presence or absence of occupants, tallying the quantity of occupants, recognizing specific individuals and tracking the movement and activity of people within a building across different time periods [13, 21]. Current studies employ various methods to collect or detect occupancy data, including CO2 sensors [22], environmental sensors [23], real-time locating systems [18], surveillance video [24], IT infrastructure [21] and passive infrared motion sensors [15]. These methods are applied across different time periods, approaches, and scales to facilitate accurate occupancy prediction. For instance, Candanedo and Feldheim (2016) employed a classification model to predict occupancy in a small office room based on environmental conditions such as temperature, humidity, light, and CO2 levels [23]. Salimi, Liu and Hammad (2019) utilized the Markov chain method to simulate zone-level occupancy and occupants’ locations [18]. Zou *et al*. (2017) proposed a real-time head detection and occupancy measurement algorithm using surveillance video within a university office setting [24].

The study of activities in offices mainly focuses on the physical activities (sitting, standing and moving) for promoting health. The common methods of capturing activities include taking snapshots [25], observation [26], using trackers and self-reported questionnaire [27]. The normal sitting time in the office counts for approximately 54% to 93% of working time [28–30], which is the major activity around the office; the percentage of time spent on standing is 11% and 5% [31]. The percentage of reported walking time is about 12% [31]. The movement behaviour is seen as the product of the spatial configuration factors, the functional attractors and the human needs [25].

Therefore, while the majority of studies focus on improving the accuracy of occupancy sensors for energy efficiency in the office environment [16, 32], there is a significant potential in the analysis and prediction of occupancy in offices with the sensor-based datasets, as it could have a further implication in the post-pandemic time to inform and utilise the office design, which is discussed in the next section.

### 2.2 Co-working space as a future office solution

The post-pandemic era has brought about a significant shift towards hybrid working, emphasizing the need for increased flexibility in choosing work locations and time. The knowledge-based industries and sectors, in particular, are expected to embrace a balanced hybrid working model where employees are not required to be present in the office five days a week [33]. As a result, anticipated office occupancy levels are smaller than the actual total office occupancy level [34], for instance, the implementation of Covid-19 restrictions led to a decrease in occupancy levels to 51% [35]. Alternative workplace options such as home offices, co-working spaces and remote workspace have gained popularity. Consequently, the role of the office is gradually shifting away from providing spaces for day-to-day work, and a fresh look at how the office space can be used to enhance well-being, collaboration, productivity, corporate culture and work experience in the future is necessary [33, 36].

To achieve this, office design needs to become more flexible, diverse and resilient by replacing fixed allocated open-plan seats and cubicles with adaptable layouts and implementing desk-sharing rules within multi-functional areas. These changes maximize space efficiency while reducing rental, maintenance, and operational costs [34]. However, demand for flexibility also leads to some challenges in office design and planning as it brings new complexities and uncertainties [37]. While hot-desking system provides high flexibility and optimises the space utilisation in a hybrid working setup, it also suffers the drawbacks like a lack of ability to personalise the space and a limited sense of ownership [38, 39]. Therefore, it is essential to explore the variation in office occupancy levels after the pandemic. Analysing and predicting the number of occupants can aid in design decisions by answering questions such as how many employees are expected to come to the office and when they typically arrive during hybrid working. This information can inform the reconsideration and redesign of facilities, seating arrangements and design styles accordingly.

Co-working spaces, with their emphasis on flexible, dynamic and resilient design, offer an alternative to home offices and semi-public spaces [40], making them an effective model for the future of work environments [6]. Some literature has explored the use, function and design style of co-working spaces. Kartika, Setijanti and Septanti [7] have identified co-working spaces as favourable workplaces for start-ups, freelancers, and creative industries. Kwiatkowski and Buczynski [41] outline five core values for co-working spaces: collaboration, openness, community, accessibility and sustainability. Users are motivated to utilize co-working spaces to separate work and personal life, achieve a better work-life balance and connect with like-minded professionals. Factors such as convenient location, open layout, shared facilities, flexible leases and knowledge sharing impact users’ satisfaction [9]. In questionnaire surveys, occupants also express a preference for minimalist and industrial design styles [7]. However, there is a lack of literature specifically focusing on the design logistics of co-working offices. By utilizing sensor-detected occupancy data and analysing activity distributions, it is possible to further explore the occupancy levels and usage of co-working spaces, providing valuable insights for evidence-based data-driven design—a facet that has not been extensively demonstrated in published research.

Overall, co-working spaces hold great potential as a future office solution, and further investigation into their design and occupancy dynamics through the lens of data-driven analysis can contribute to the advancement of workspace design and utilization. Therefore, this study attempts to investigate the temporal occupancy variations, zone-based occupancy distribution and seat utilization rates and identify the spatial hotspots for users’ occupancy and activities.

## 3 Data and methods

**Fig 1:**
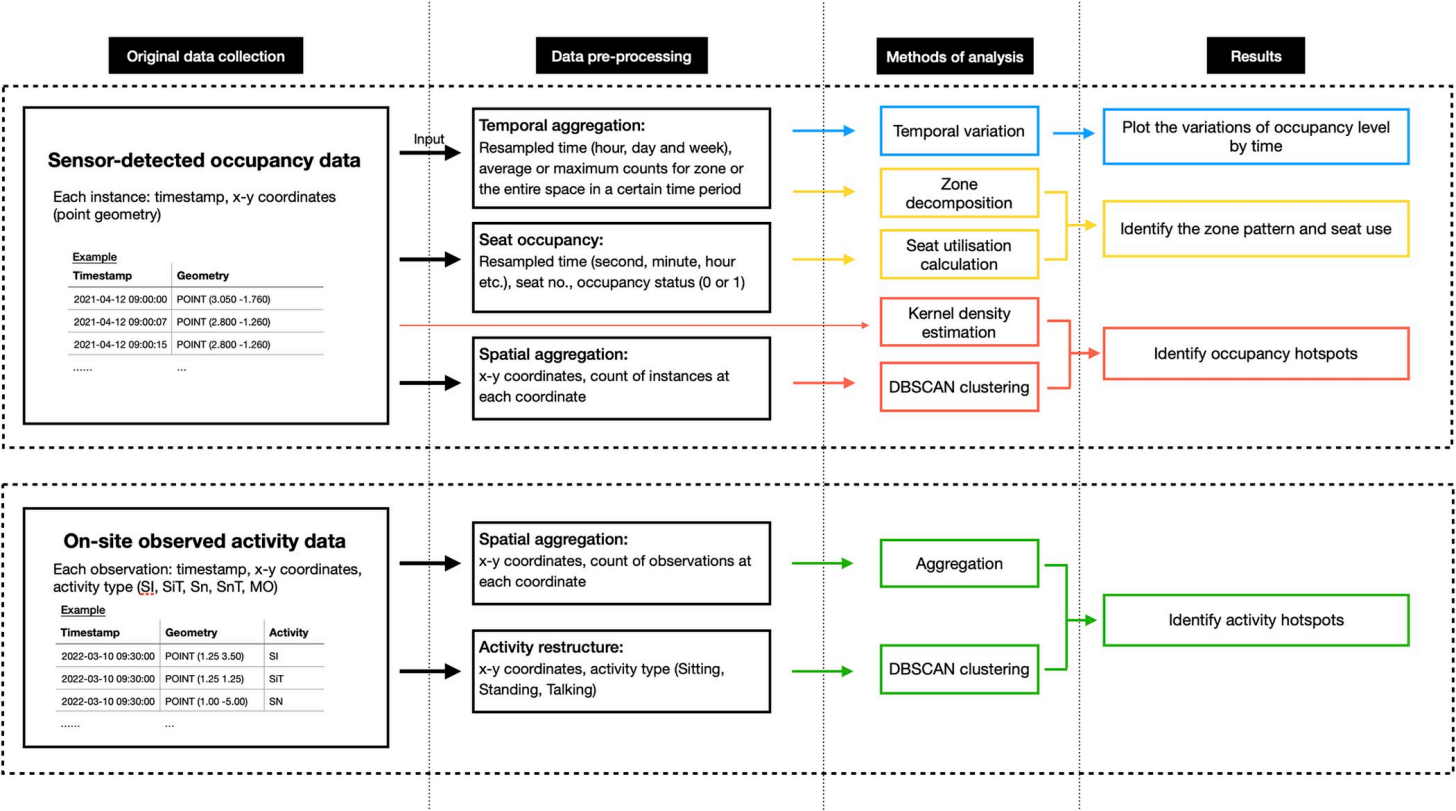
Methodological framework.

This section provides an overview of the study site, the data collection process, and the analytical tools employed in the exploratory analysis. The method framework is presented in Fig 1, outlining four main steps: original data collection, data pre-processing, analytical methods and result production. Examples of the data structure for both the original and transformed data are provided, along with a brief overview of the applied methods (introduced in Section 3.2) and the corresponding outputs (discussed in more detail in Section 4).

### 3.1 Study site information and data collection

#### 3.1.1 Site information

The case study in this manuscript examines a dynamic co-working office space located near Old Street in London, England, which opened for operation in early 2020. This space is designed to promote multi-functionality, adaptability and creativity, offering not only a collaborative workspace but also serving as a local café, event venue and exhibition space. With a maximum capacity of approximately 80 individuals, the co-working office operates on weekdays from 9 am to 6 pm.

**Fig 2:**
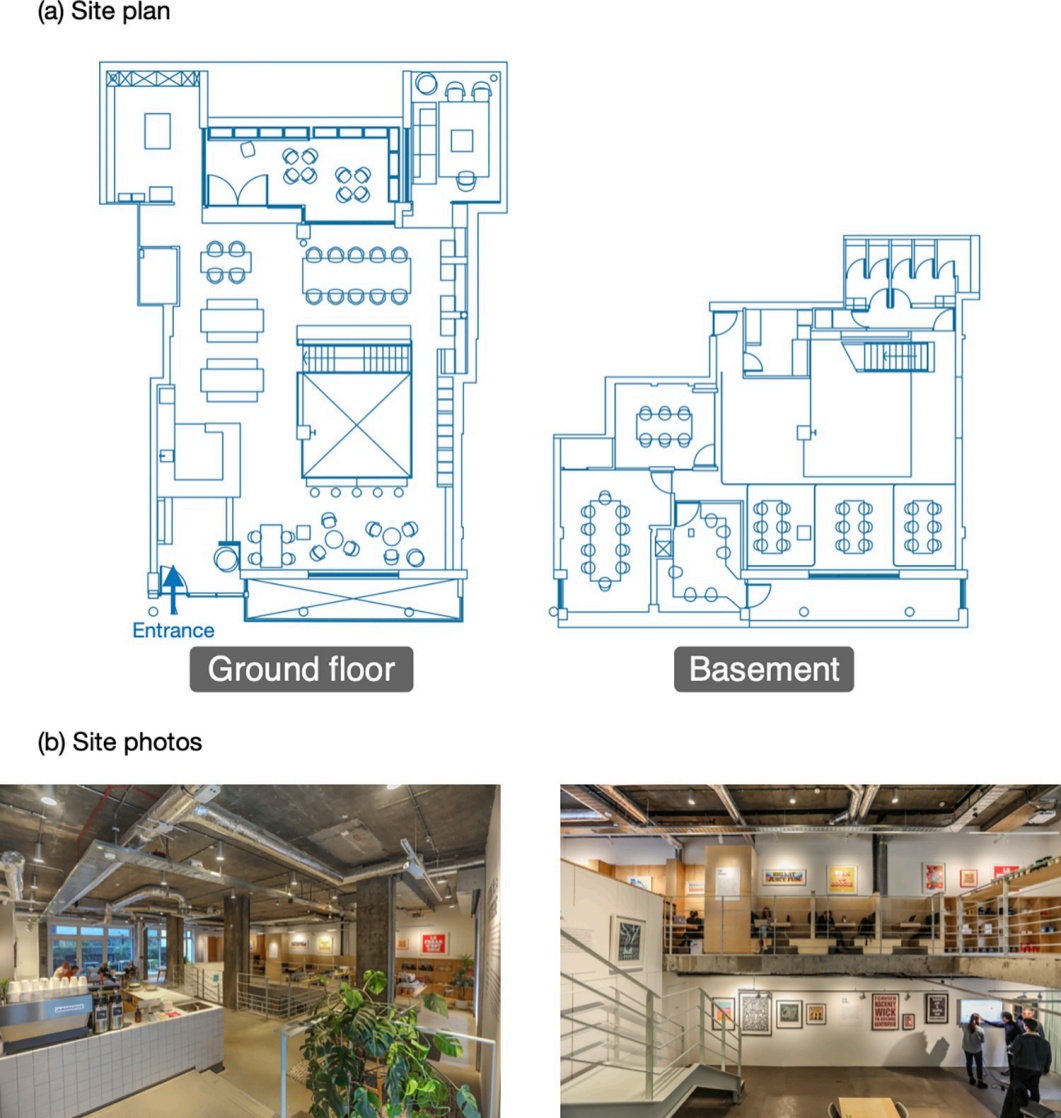
Basic site information.

Fig 2 provides an overview of the case study site, which encompasses both the ground floor (left side in Fig 2A) and the basement (right side in Fig 2A) of the building. The ground floor includes a reception area, a café and various seats, while the basement primarily functions as meeting areas, featuring two enclosed meeting rooms and flexible tables for both group meetings and individual work. Fig 2B includes photographs that capture the ambiance and layout of the co-working space as a visual depiction of the site.

#### 3.1.2 Data collection

Two datasets were collected for this study: sensor-tracked occupancy data and on-site observed activity data, and the data collection period is shown in Table 1. The collection time covers the large seasonal variations in the country. The sensor-tracked data is directly obtained and shared by the lab_ collective through data exchange. The access to the site for on-site data collection is also permitted by the lab_ collective. All collected data are fully anonymized, ensuring the privacy and confidentiality of individuals without any possibility of personal identification or association.

**Table 1:** Data collection period.

	Data collection period	Duration
Sensor-tracked occupancy	Mid-April 2021 to early February 2023	~1 year and 9 months
On-site recorded occupants’ activity	March 2022 to February 2023	1 year

**Fig 3:**
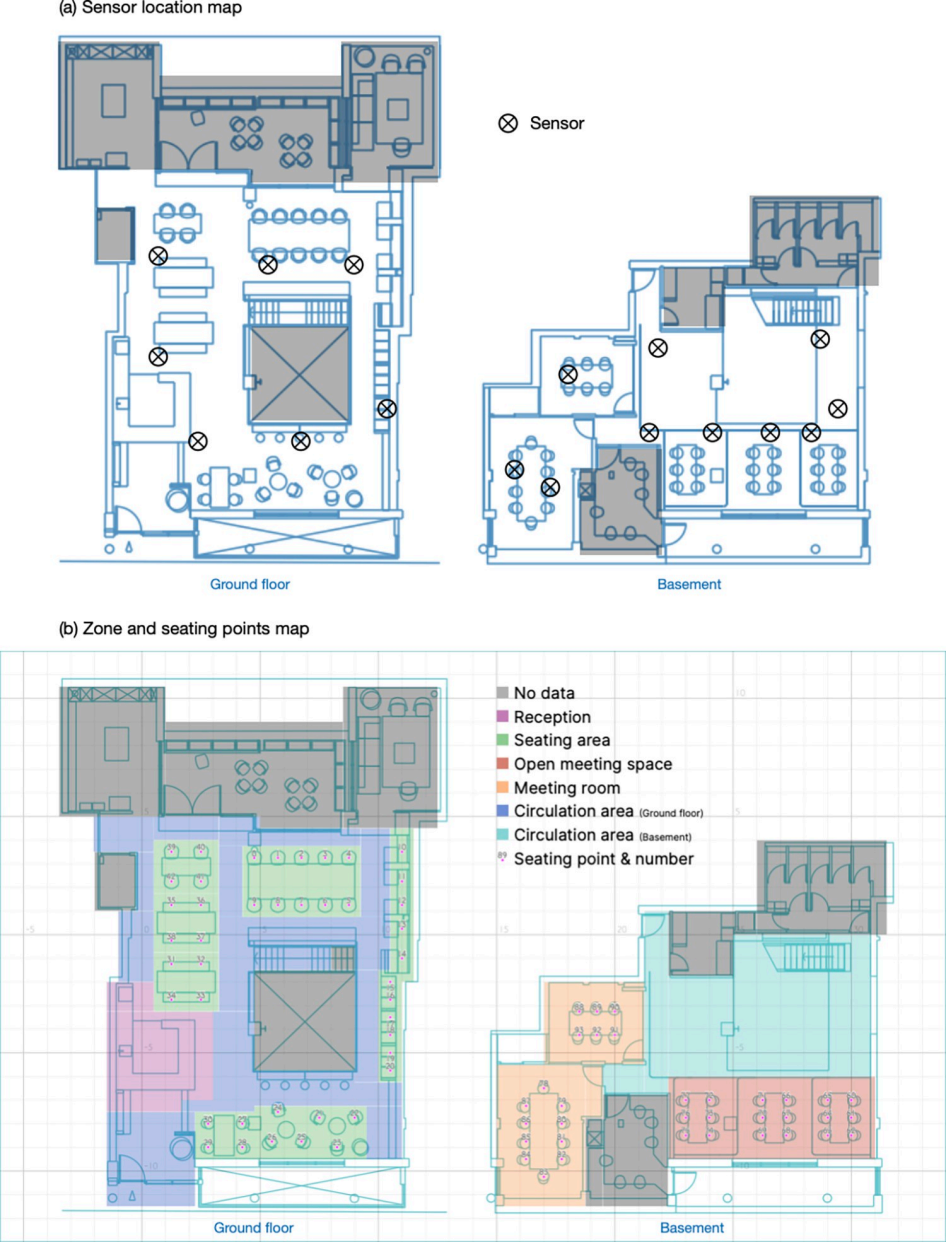
Additional site information map.

*Dataset 1*: *Sensor-tracked occupancy*. The occupancy data was obtained using PointGrab sensors installed on the ceilings within the co-working space (location indicated in Fig 3A). These sensors, specifically designed for space management and workplace optimization in hybrid working environments, offer high spatial and temporal resolution for detecting the presence of occupants. They do not track movement or collect individual identity information.

The sensors capture the timestamped location of each occupant in an x-y coordinate system at one-second intervals, generating instances of data. The resolution of the sensors is sufficient for space and design utilisation purpose [13]. The data collection period spanned from mid-April 2021 to early February 2023, covering approximately one year and nine months. The original data was stored in PostgreSQL [42].

**Fig 4:**
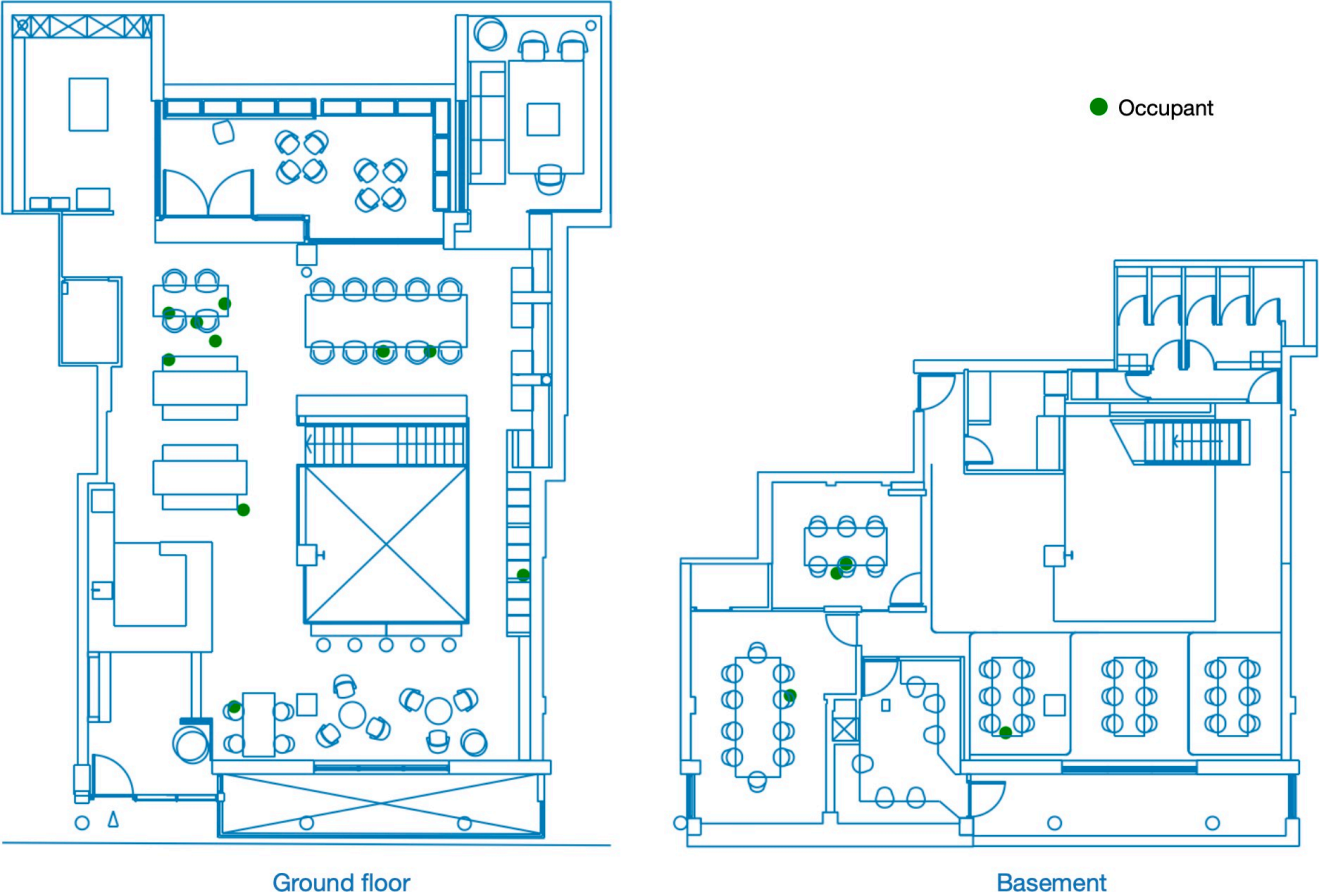
Example of occupancy snapshot. Timestamp: 2022-11-08 14:32:00.

The raw data covering the entire timespan are simplified through a data cleaning process which selects data based on its timestamp and coordinates. Firstly, only the data in working hours (9:00 to 18:00) in weekdays (Monday to Friday) are retained for analysis; Secondly, several areas are excluded, such as toilets, storage spaces and private spaces that are not accessible by all occupants (as indicated in Fig 3(B)). As a result, the dataset consists of over 13.4×10^6^ unique timestamps, covering 477 business days, with more than 145×10^6^ sets of coordinates representing the occupants’ locations at each timestamp. An example of an occupancy plot is depicted in Fig 4. The data was processed in Python 3.11 [43] with the packages pandas [44], geopandas [45], SciPy [46] and scikit-learn [47].

*Dataset 2*: *On-site recorded occupants’ activity*. In addition to the sensor-tracked occupancy data, on-site manual observation was conducted to capture occupants’ activities within the co-working space to determine how space is used [48]. Manual observation provides supplementary information to the sensor data [49]. Occupants’ activity level refers to the measure of engagement and actions performed within a specific space or environment, typically expressed as the frequency or intensity of various activities conducted in that area.

**Fig 5:**
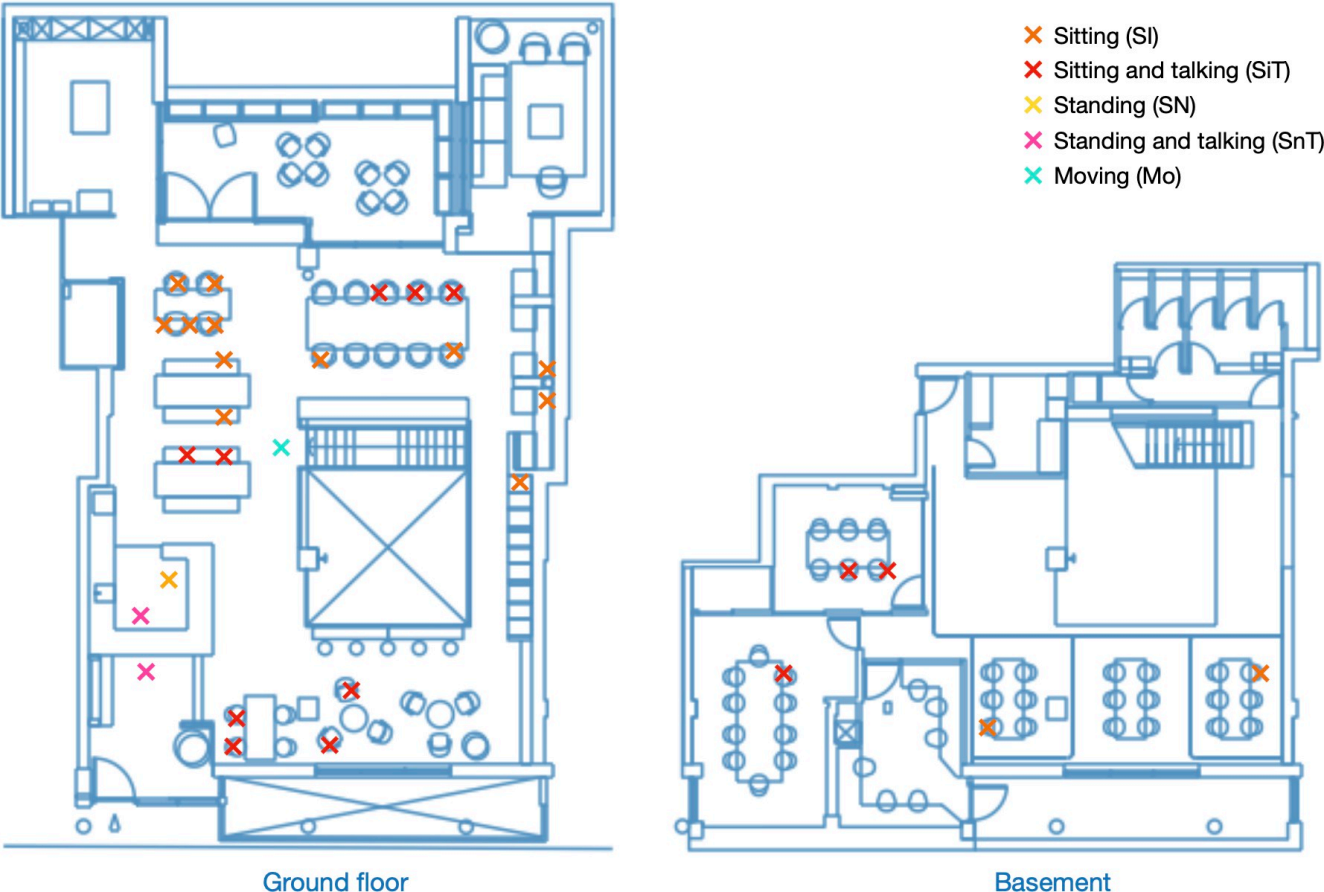
Example of activity snapshot data. Timestamp: 2022-11-08 14:30:00.

The on-site observation in this study spanned 13 days over a twelve-month period, from March 2022 to February 2023. Data collection took place on one designated day each month, except for March 2022, which included two days as part of a pilot observation. The observer documented occupancy and activity data on an hourly basis from 9:30 to 17:30 during typical observation days, with minimum awareness from occupants. The observer was positioned in unobtrusive locations and did not approach or interact with the occupants during the data collection to avoid influencing their behaviour or drawing their attention. In total, 110 snapshots were taken, capturing 1902 individual observations. A snapshot provides a comprehensive view of the entire space at a specific timestamp, while an observation represents the location coordinates of a single occupant along with their corresponding activity information. Activities were categorized based on direct observation, resulting in five identified types: ’sitting’ (SI), ’sitting and talking’ (SiT), ’standing’ (SN), ’standing and talking’ (SnT), and ’moving’ (MO). It is important to note that, due to the increased use of remote working tools, individuals observed ’talking’ may be engaged in various situations, such as conversing with nearby colleagues, presenting and reporting to leaders, or participating in online meetings via video conferencing tools. Fig 5 provides an example snapshot recorded during the observation period.

**Fig 6:**
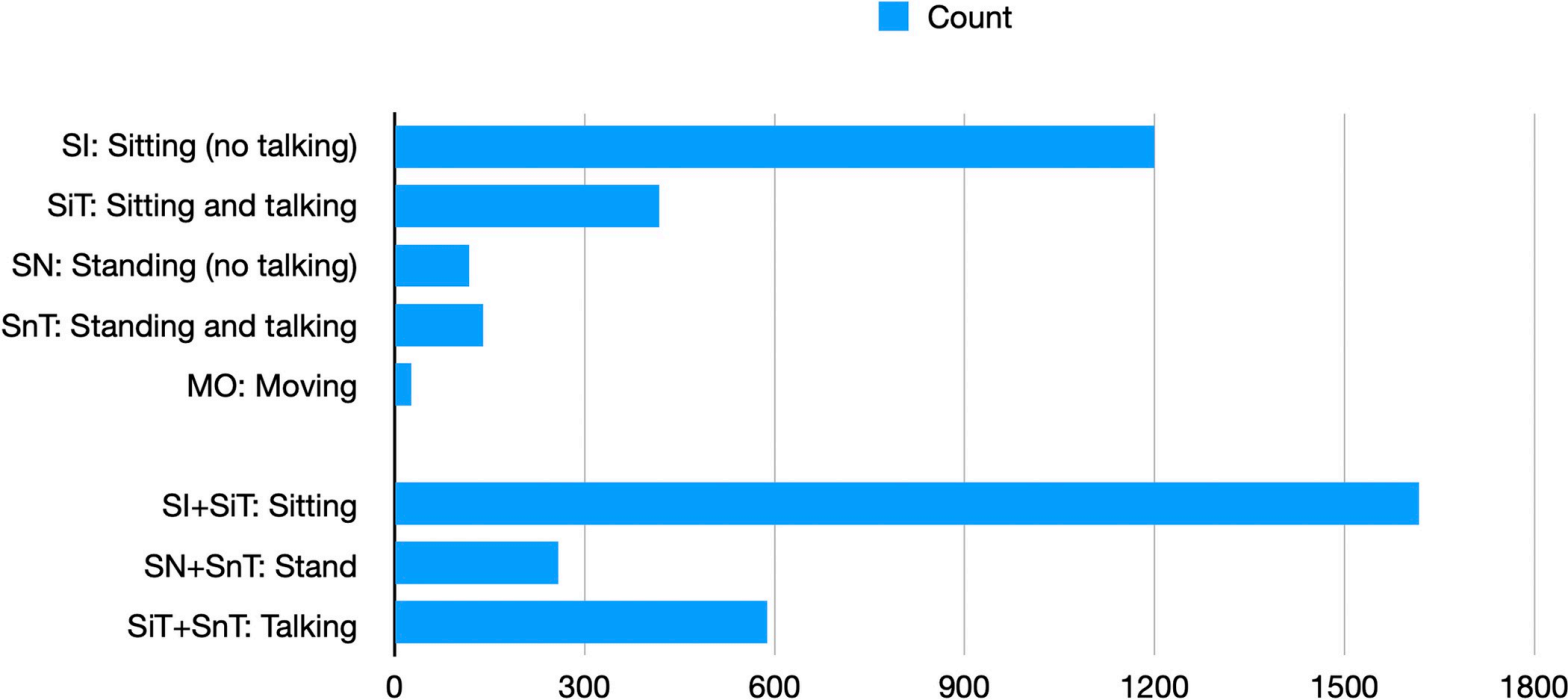
Breakdown of the counts for different activity types.

In this dataset, the majority of occupants were observed to be ’sitting’ (n = 1,200, 63.1%), followed by ’sitting and talking’ (n = 418, 22.0%). The categories of ’standing and talking’ and ’standing’ accounted for smaller proportions, with 7.4% (n = 140) and 6.2% (n = 118) respectively. The category of ’moving’ had the lowest occurrence, comprising only 1.4% (n = 26) of the observations. The data was further restructured into three key types: Sitting (SI and SiT), Standing (SN and SnT), and Talking (SiT and SnT). The breakdown distribution is depicted in Fig 6.

### 3.2 Spatial-temporal analysis

Spatial-temporal analysis is a widely utilized method in geospatial analysis that involves the processing and examination of data incorporating both time attributes and absolute and relative positions in three-dimensional space [50]. In a dataset that incorporates both spatial and temporal dimensions, an event refers to a phenomenon that occurs at a specific time and place [51]. It represents a combination of spatial and temporal attributes at a particular moment. While the application of this analysis in the interior environment is relatively less common compared to other scenarios like urban transport, tourism, climate environment and public health [52–55], there exists significant potential for employing this method to analyse spatial and temporal patterns of human behaviour within indoor environments, as demonstrated in this study.

#### 3.2.1 Temporal variation of the occupancy level

The occupancy level refers to the count of individuals present within a building, expressed as a peak or average percentage and commonly measured over various time periods [56]. In this study, the occupancy data is analysed descriptively to examine the temporal variation of the occupancy level. This involves plotting the occupancy level against time, including the maximum occupant counts for each day and the average occupancy level by hour, business day and month. The number of occupants at each timestamp is counted by summing up the number of coordinates recorded in the dataset. The frequency of this dataset is then transformed from seconds to hours and business days. The maximum number of people per day is computed as the daily occupancy level.

#### 3.2.2 Spatial use decomposition and seat utilisation evaluation

Service and facility decomposition is applied to determine the need of the space. Typically, the area in a public facility is divided to primary, amenity, circulation and technical areas to perform decomposition, while the activities in the facility are characterised by the duration, number of users and area per person [48]. Spatial use decomposition is applied in this study to determine the functional zones within the co-working space. The space is decomposed into several zones, including the working area, transitional area, reception area and meeting rooms (as illustrated in Fig 3(B)). The presence of occupants in these functional zones is aggregated over time to analyse the spatial use patterns. The space utilisation rate is calculated by the occupancy over the footprint. Flexible working zone, also noted as seating area, is the hot-desking seats that people can freely access and sit down for working. The reception zone is a café reception and its waiting area where the public can order drinks and food. Meeting zones have two sub-categories, one is the enclosed meeting rooms and the other is the open meeting tables which could also be used for sit-down working. The remaining areas are categorised as circulation areas on the ground floor and basement.

Further to the zone level, the utilisation level of the seating spaces is evaluated. Seat utilization refers to the average duration of time that a specific seat is occupied for various purposes [56]. In a previous study, the utilization level is represented as the percentage of occupied time over total time, at minute and hour levels [49]. In this study, the seat utilization calculation is performed at multiple levels, including the second, minute, quarter, and hour levels, based on the high-resolution occupancy detection data. Each seat is assigned with an index number (seat_id), ranging from 0 to 42 on the ground floor and 60 to 93 in the basement (also shown in Fig 3(B)). A seat is considered ‘occupied’ if an occupant is detected within a 0.5 metre radius around the seat at any time instance.

#### 3.2.3 Point pattern analysis

Point pattern analysis is a branch of spatial analysis that focuses on studying the spatial arrangement and characteristics of a set of points or events within a geographic space. It mainly encompasses density-based and distance-based approaches [57]. This study attempts to assess the first-order property of the point pattern using kernel density estimation and the second-order property through spatial cluster analysis.

*Aggregation*. Aggregation is a process of combining data to identify spatial or temporal patterns by summing the number of instances or observations to a coarser spatial or temporal form [58]. A higher aggregation value indicates the concentration of events.

*Kernel density estimation*. Kernel density estimation (KDE) is a statistical method used to estimate the probability density function of observed data [59], providing a visual representation of point or polyline concentration. It computes the localised density estimate of features by assigning a magnitude per unit area, which represents the density of features within a given neighbourhood surrounding each feature [57, 60]. The calculation of KDE involves assigning weights to the distances of observed data points for each position. When there are more nearby points, the estimation value increases, indicating a higher probability of encountering a point at that specific location [61]. A grid structure is usually generated to represent the density values.

The predicted density at a new coordinate (*x*, *y*) is represented in Eq 1 [62]

$$Density(x,y)=\frac{1}{{\left(radius\right)}^{2}}\sum_{i=1}^{n}\left[\frac{3}{\pi }∙{pop}_{i}{\left(1-{\left(\frac{{dist}_{i}}{radius}\right)}^{2}\right)}^{2}\right]$$ (1)

$$\mathrm{for}\;{\mathrm{dist}}_{i}<\mathrm{radius}$$

where *i* represents an index used to denote individual data points; pop_*i*_ is an optional parameter representing the population field value of point *i*; dist_*i*_ measures the distance between point *i* and a point with coordinate (*x*, *y*). In the case of analysing occupancy level, the more frequent occurrence of occupancy around a coordinate result in a higher density of occupancy.

*Unsupervised machine learning*: *Spatial clustering*. Spatial cluster analysis is an exploratory machine learning tool for gaining a greater understanding of a dataset [52], to uncover the spatial relationships and reveal areas of concentrated or dispersed occurrences. Density-Based Spatial Clustering of Applications with Noise (DBSCN) is the unsupervised learning clustering technique that identifies the connected area with a high concentration of points, separated from other clusters with a lower point density [63]. The algorithm chooses a random point *p* at the beginning and identify all density reachable points from p based on two hyperparameters (epsilon value and minimum points), then determine whether p is a core point or a border point. A cluster is established if p is a core point, while next point in the dataset is visited if p is a border point. The process is repeated until all points are visited. We consider DBSCAN suitable for this analysis as it computes the distance between points and cluster the neighbouring points. It has the advantages of automatically detecting and ignoring noisy points, while detecting cluster points that are not linearly separable [64]. This clustering analysis is applied on both occupancy and activity data, in order to identify the spatial clusters. This analysis is implemented through scikit-learn [47, 65]. The optimal parameter values, epsilon value (eps) and minimum samples (min_samples), are determined by iterating through different combinations and selecting the combination that results in a manageable number of clusters and an acceptable silhouette score. For the occupancy dataset, a three-dimensional set is used, consisting of the x and y coordinates and the count of instances at each coordinate set. For the activity dataset, only the x and y coordinates are used as input data for the cluster analysis.

## 4 Results

### 4.1 Spatial-temporal variation of occupancy level

#### 4.1.1 Descriptive data

**Fig 7:**
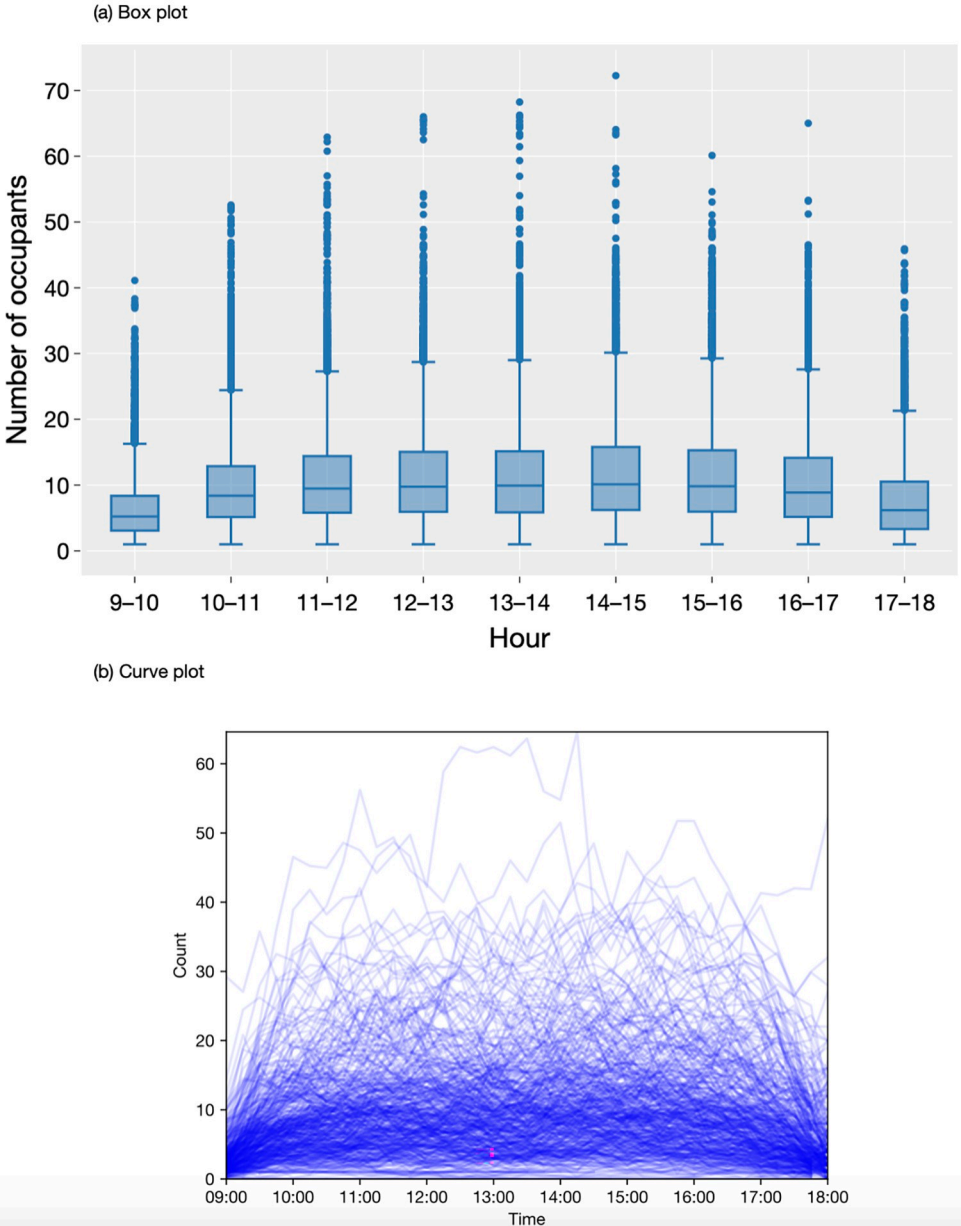
Variation of hourly occupancy level.

**Fig 8:**
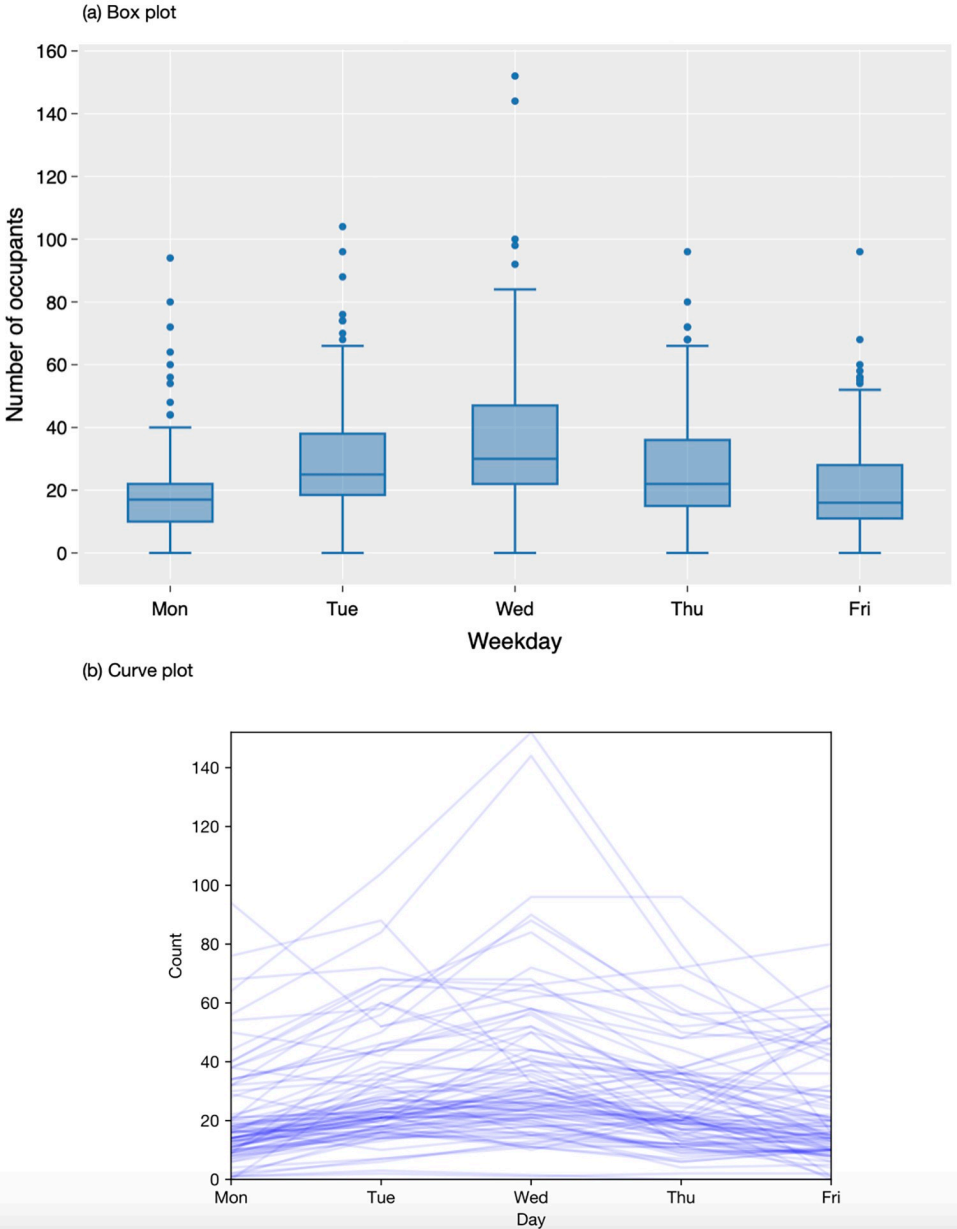
Variation of daily occupancy level in a week.

**Fig 9:**
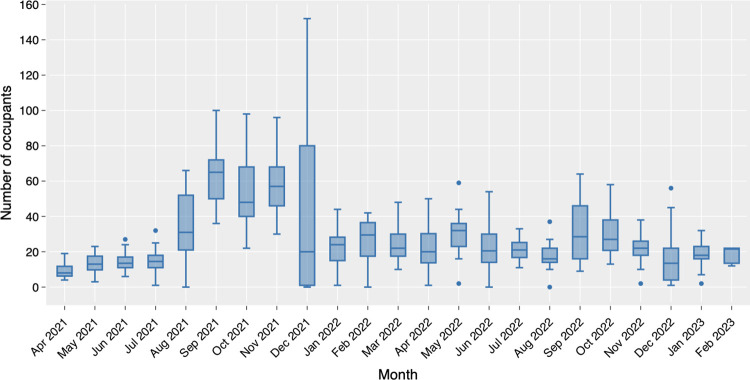
Variation of daily occupancy by month.

**Fig 10:**
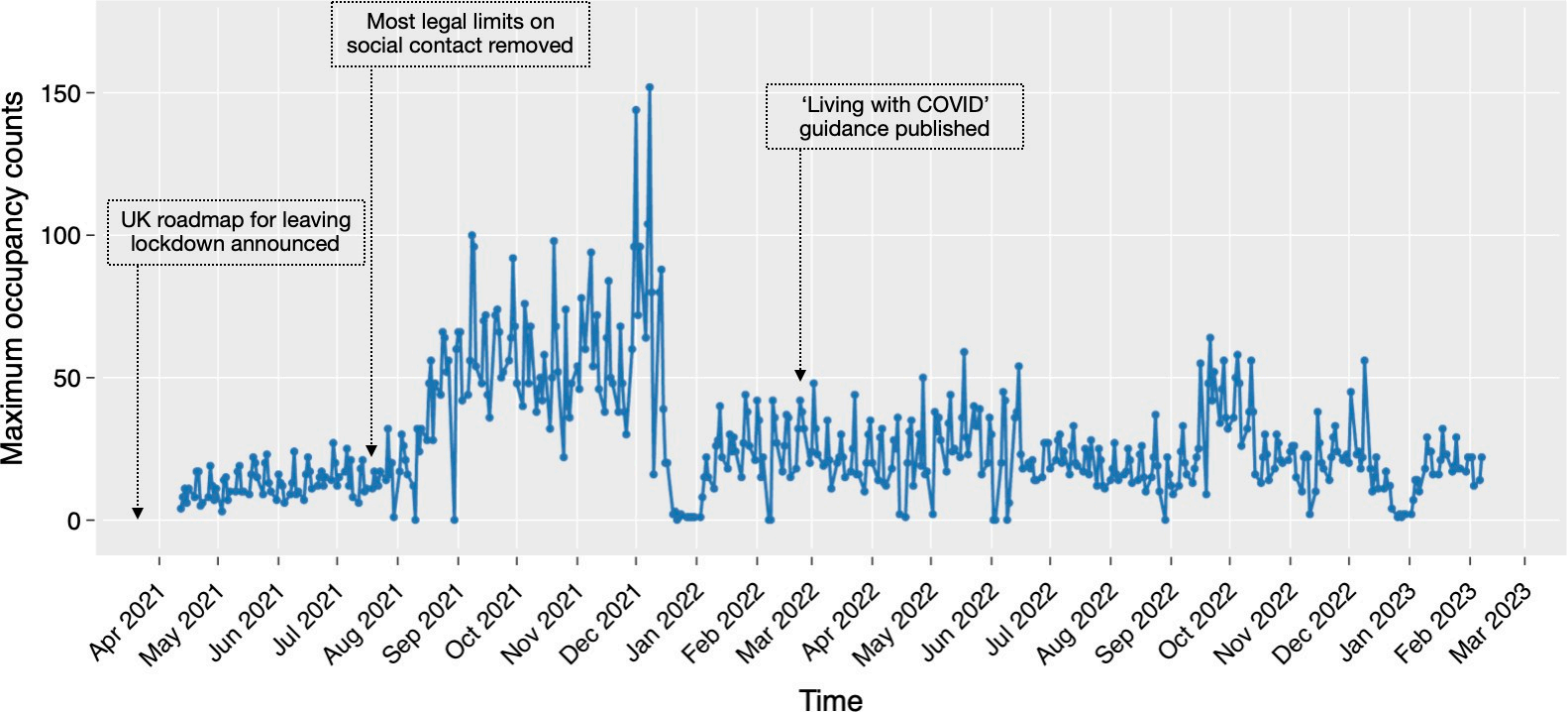
Daily maximum occupancy over the collection period.

The descriptive analysis of the collected sensor data is presented in Figs 7–10. The box plots show the variation of hourly average occupancy level (Fig 7(A)), daily average occupancy level in weekdays (Fig 8(A)) and daily average occupancy level in different months (Fig 9). The curve charts present the variation of occupancies in a day and a week by plotting out the variation of all instances. On a typical day, the occupancy level shows a steady increase from the beginning of the day at 9am, reaching its peak at lunch time around 1pm to 3pm, and then gradually declining after 5 pm. Wednesday appears to be the busiest day of the week, with a higher number of occupants compared to Monday and Friday, which have relatively lower occupancy levels. The daily number of occupants varies significantly across the months. An increase in occupancy level is observed in August 2021, while December exhibits a substantial variation with an extremely high number of occupants and some relatively low values. The autumn months (September, October, and November) tend to have higher occupancy levels compared to other months. Overall, the occupancy level remains relatively stable between 20 to 40 occupants throughout 2022 and early 2023.

Fig 10 depicts the time plot of the daily maximum occupancy series. The general trend aligns with the findings from the monthly occupancy plot. However, the day-to-day fluctuation suggests a seasonal cycle in the data. A previous study has conducted time series analysis and applied ARIMA modelling to predict the daily maximum occupancy level. A seasonal lag of five days were identified in the dataset, corresponding to the rotation of weekdays [66].

#### 4.1.2 Space decomposition and seat utilisation

**Fig 11:**
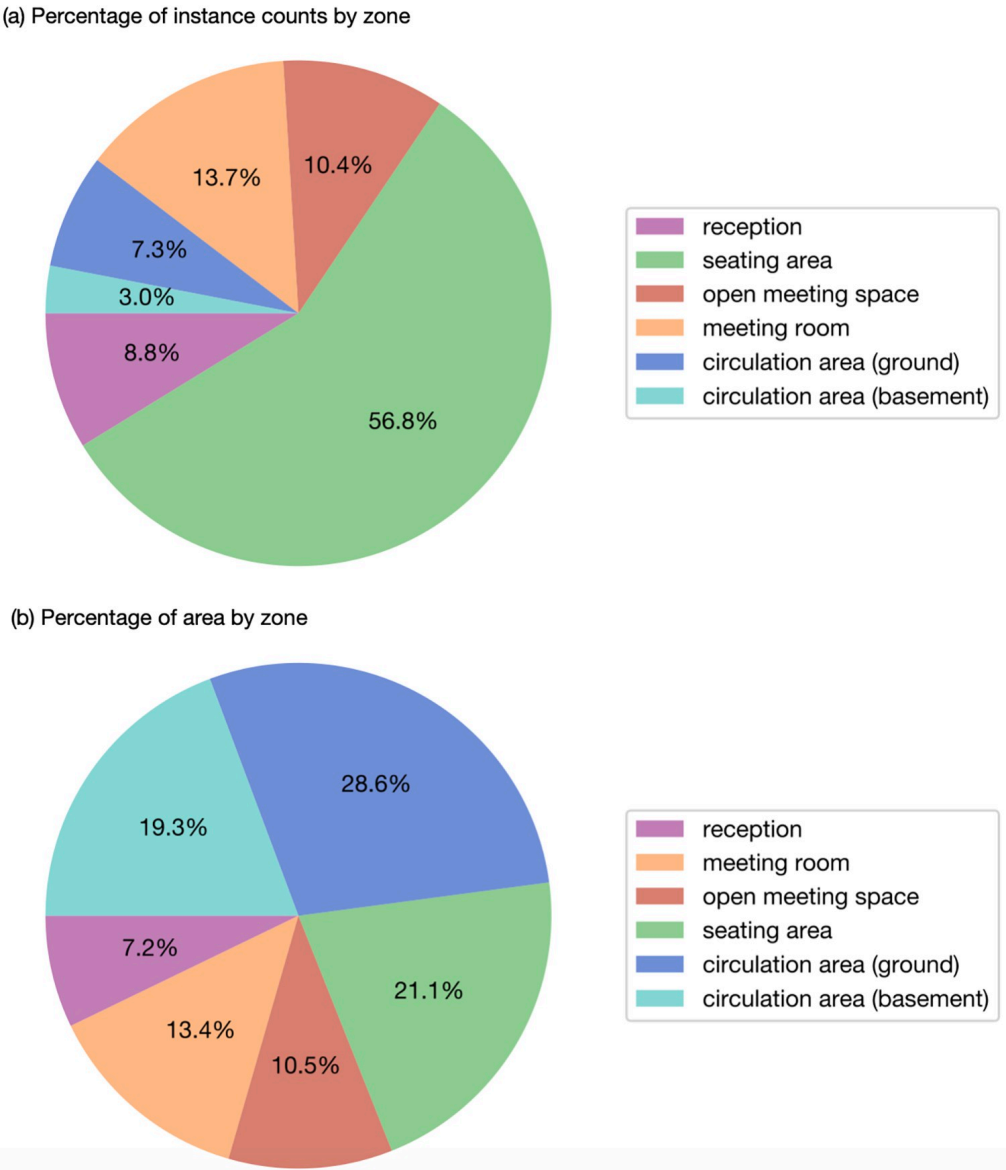
Percentage of occupancy and area by zones.

This section presents the results of decomposition by zone and the calculation of seat utilisation rates. The percentage of instance counts in each zone over the whole time period is shown in Fig 11. The seating area is the most frequently used zone, accounting for 56.8% of the counts. Meeting rooms and open meeting spaces follow with 13.7% and 10.4% respectively. The reception area occupies 8.8% of the counts, while circulation areas take 10.3% in total, with ground floor circulation area at 3.0% and 7.3% respectively. The calculation of occupancy to area ratio in Table 2 indicates the space utilisation rate by zone, showing that the mostly utilised zone is seating area and reception.

**Table 2:** Space utilisation rate by zone.

	% of occupancy	% of area	Space utilisation rate (occupancy instance per unit area)
**Reception**	8.8%	7.2%	1.22
**Seating area**	56.8%	21.1%	2.69
**Open meeting space**	10.4%	10.5%	0.99
**Meeting room**	13.7%	13.4%	1.02
**Circulation area (ground)**	7.3%	28.6%	0.26
**Circulation area (basement)**	3.0%	19.3%	0.16

**Fig 12:**
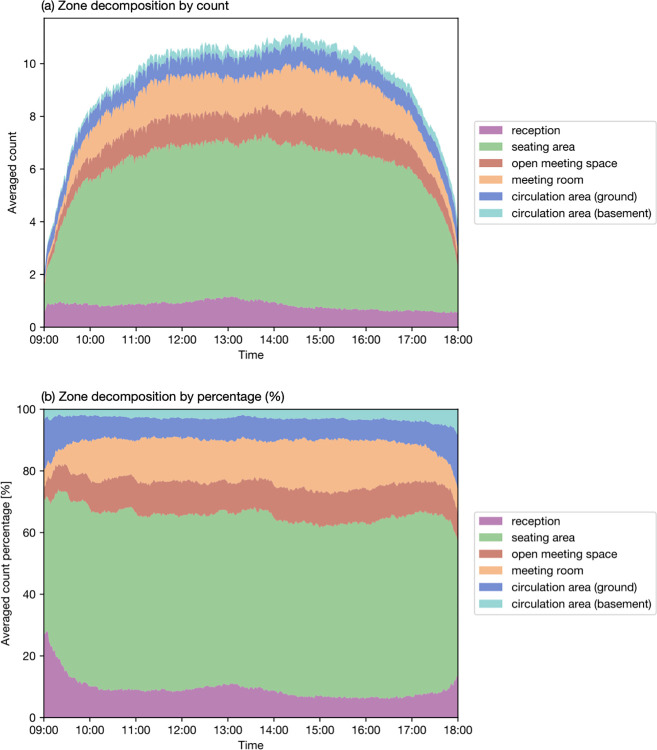
Space occupancy decomposition by zone.

Fig 12 illustrates the variation in zone usage throughout the day. Fig 12(A) shows the averaged counts, while Fig 12(B) presents the percentage values. The reception area maintains a relatively stable occupancy with approximately one person throughout the day. During lunchtime (12:30 to 13:30), the number of people slightly increases, indicating the presence of more than one person at times. Other occupants typically arrive and use the working area from 10:00 to 17:00. The variations in occupant distribution in the seating area and open meeting space are aligned. The peak use of meeting rooms occurs in the afternoon, around 14:30 to 16:30. More occupants are found in circulation areas at the beginning and end of the day as they enter and leave the space.

**Fig 13:**
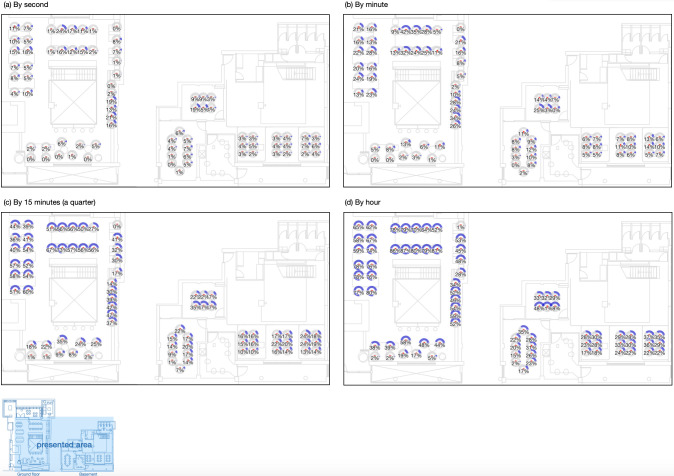
Seat utilisation rate by different granularity levels.

Seat utilization rates are calculated as the percentage of occupied time over the total time, as shown in Fig 13. Four different time granularities are considered: second (Fig 13(A)), minute (Fig 13(B)), quarter/15 minutes (Fig 13(C)) and hour (Fig 13(D)). The results reveal significant differences when the calculations are made at various granularity levels. The average utilisation rate is 6%, 13%, 29% and 43% at second, minute, quarter and hour levels respectively. At the second level, the highest utilization rate is 24%, while the rates increase to 42% at the minute level, 73% at the quarter level, and 87% at the hour level. Table 3 displays the top 10 most utilized seats at each granularity level, indicating that Seats 1, 19, 8 and 9 have higher utilization rates in general.

**Table 3:** Top 10 utilised seats at different granularity (second, minute, 15 minutes, hour).

	Second		Minute		15 Minutes		Hour	
Rank	Seat No.	Utilisation rate	Seat No.	Utilisation rate	Seat No.	Utilisation rate	Seat No.	Utilisation rate
1	1	24.33%	1	41.63%	8	72.55%	8	86.94%
2	19	21.21%	2	34.55%	9	66.76%	9	86.40%
3	17	19.26%	19	33.57%	1	66.10%	5	81.48%
4	93	18.92%	8	32.09%	33	59.91%	7	79.73%
5	36	17.73%	3	28.04%	31	57.60%	33	79.69%
6	2	17.43%	17	27.93%	7	57.17%	1	79.26%
7	20	16.43%	36	27.86%	38	56.97%	6	78.58%
8	8	15.86%	20	25.65%	2	56.46%	0	77.90%
9	35	15.06%	93	25.34%	5	55.76%	31	77.81%
10	92	14.87%	6	25.26%	6	55.69%	38	77.81%

### 4.2 Identification of spatial hotspots

#### 4.2.1 Occupancy hotspots

**Fig 14:**
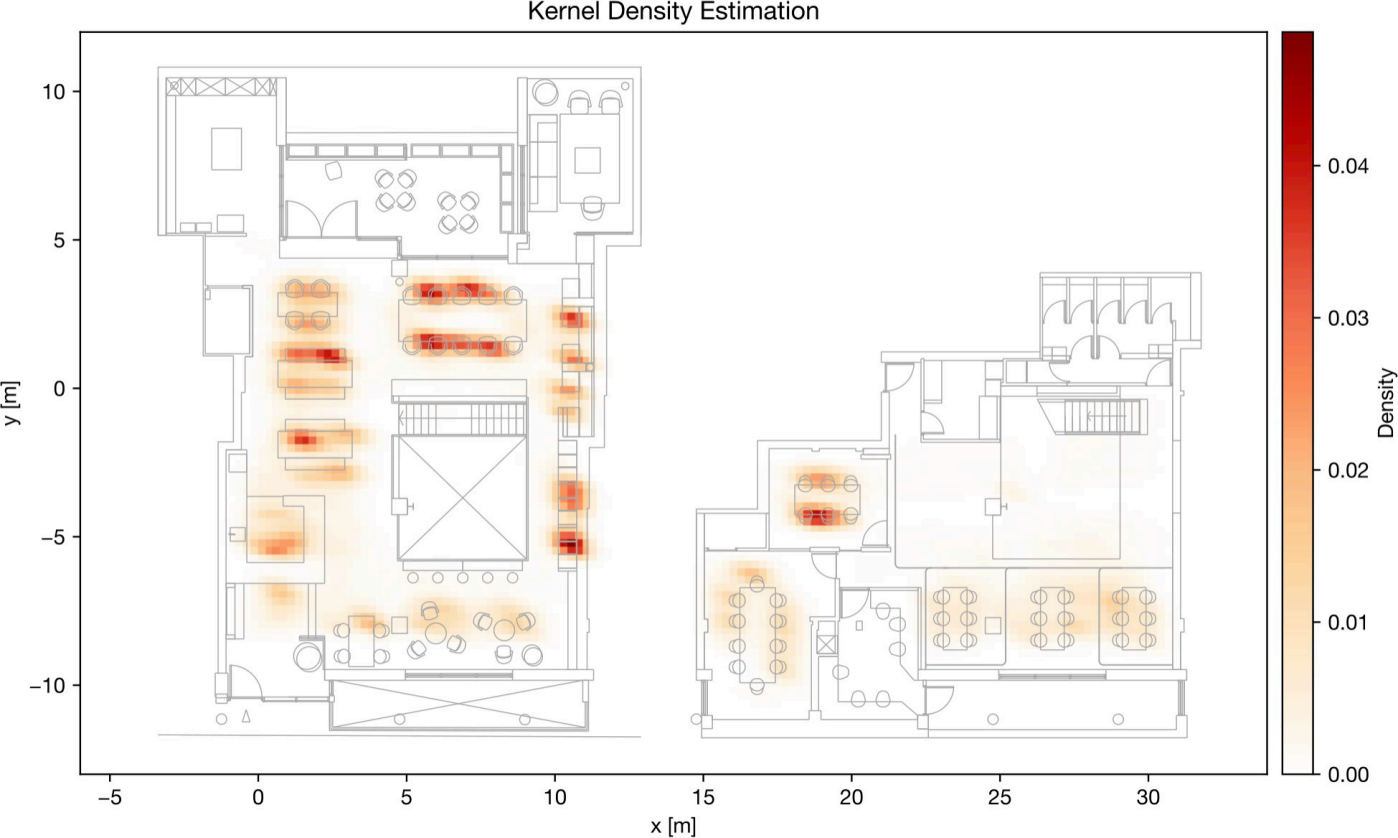
Kernel density estimation heatmap for occupancy level.

*Results of Kernel density estimation*. The kernel density estimation applied to the occupancy data reveals the concentration of occupancy in certain areas, represented by density-based features. By estimating the underlying probability density function, density values are calculated for different locations. The results are visualized in a grid structure, as shown in Fig 14. The kernel density estimation highlights several hotspots with high-density areas of occupancy, such as the table on the ground floor by the window (seats 0–9), the semi-enclosed seats on the side (seats 17–20 and 11–13), the reception area and tables near the reception (seats 31, 35, and 36). Additionally, the smaller enclosed meeting room in the basement exhibits relatively high occupancy density.

**Fig 15:**
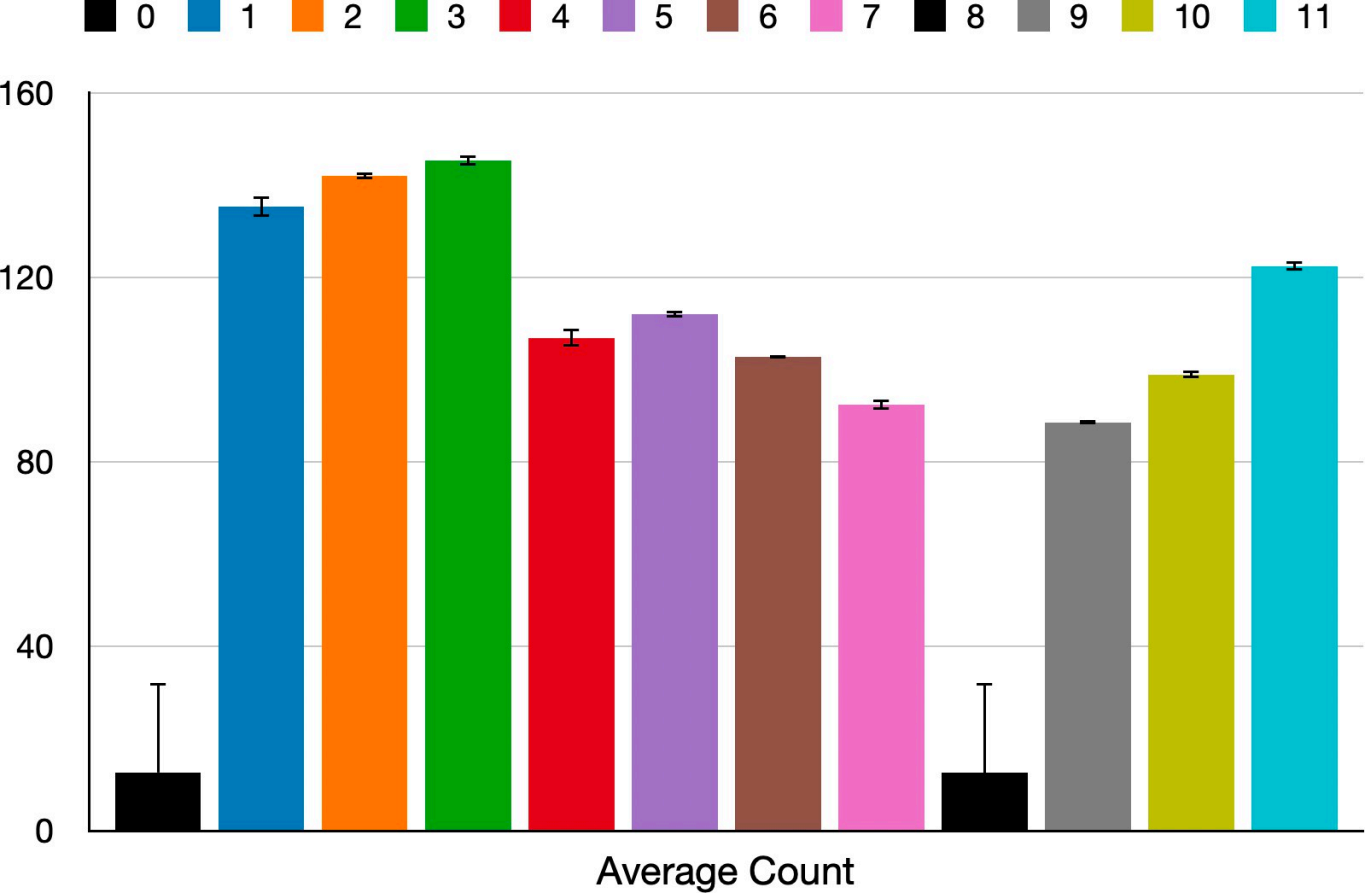
Average count of each cluster.

*DBSCAN cluster analysis of occupancy data*. DBSCAN cluster analysis is performed on the three-dimensional occupancy dataset, which includes the x-coordinate, y-coordinate and the count of instances in each coordinate set. The applied values for the parameters eps and min_samples are 2.0 and 200 respectively, resulting in the identification of 12 clusters and a noise cluster. It is worth noting that the sampled silhouette value is relatively low at 0.258, obtained from a sample size of 100,000 out of the total 1,253,946 input sets. This lower silhouette score may be attributed to the large dataset. It’s important to consider that achieving a high silhouette score may lead to either an excessive number of clusters or an insufficient number of clusters to capture any significant spatial pattern. The details of each cluster, including the cluster size, average count and standard deviation of count, are presented in Table 4, while Fig 15 visualises the average count of each cluster with standard deviation.

**Table 4:** Descriptive data for occupancy clusters.

Cluster	Cluster Size	Average Count	Total Count	Min Count	Max Count	Count std
-1	78136	1662.82	129925963	17	680749	13409.50
0	643770	12.62	8126753	1	130	19.35
1	662	135.38	89620	132	139	2.21
2	202	141.97	28677	141	143	0.76
3	287	145.39	41726	143	147	1.12
4	591	106.88	63164	104	110	1.98
5	209	112.014	23411	111	113	0.81
6	84	102.83	8638	102	103	0.37
7	329	92.45	30417	90	94	1.10
8	529124	12.56	6644163	1	122	19.48
9	125	88.60	11075	88	89	0.49
10	166	98.97	16429	98	100	0.82
11	261	122.43	31955	121	124	1.05

**Fig 16:**
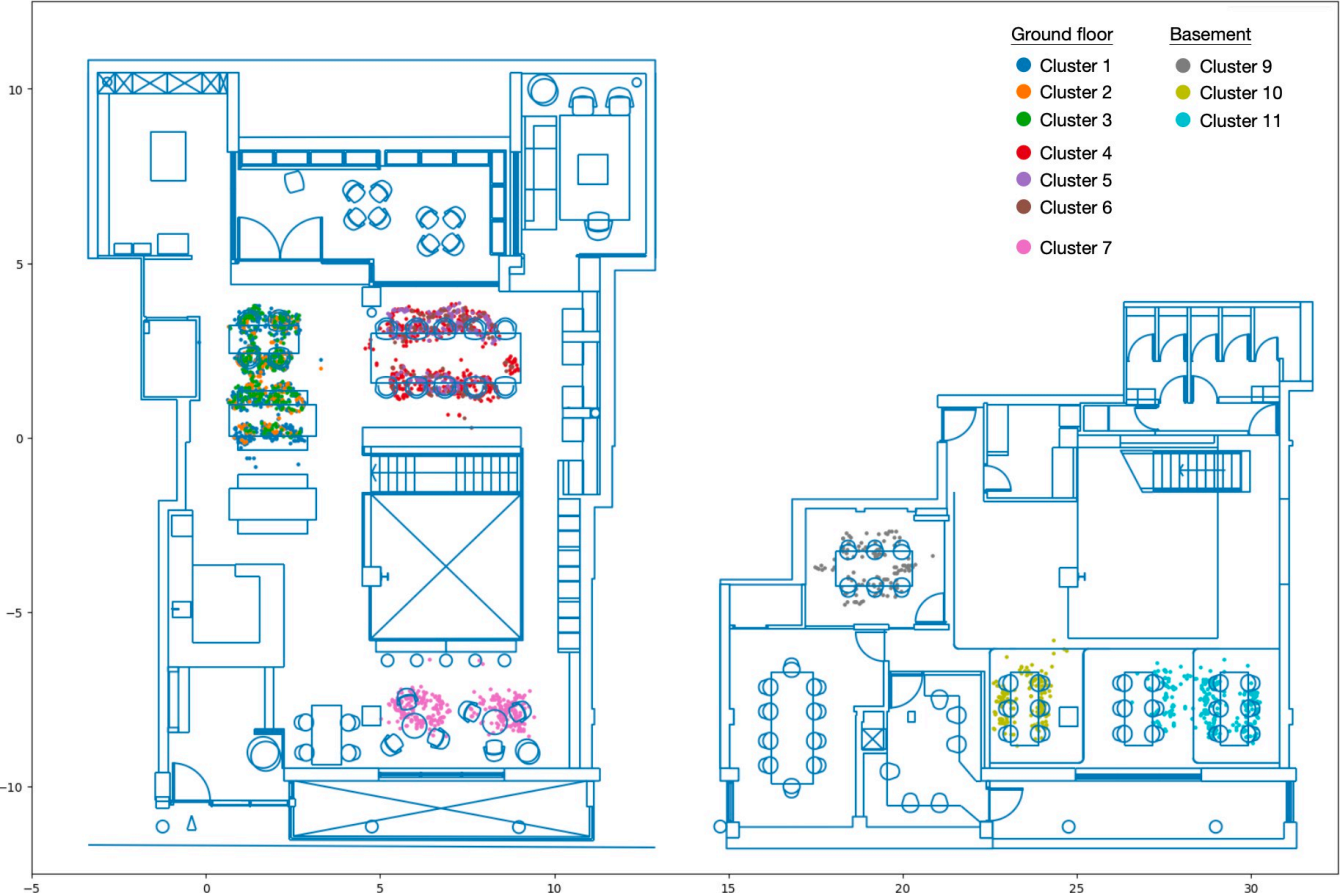
DBSCAN clusters of occupancy level.

After excluding the two largest clusters with very low average counts and high standard deviations (cluster 0 and 8), the remaining clusters reveal areas with high occupancies, as depicted in Fig 16. The identified hotspots include three main areas on the ground floor: the two smaller tables on the left-hand side (seats 35 to 42), the large table near the window (seats 0 to 9) and the sofa seats near the entrance (seats 21, 22, 24 and 25). In the basement, the clusters are found in the smaller enclosed meeting room (seats 88 to 93) and around the two open meeting tables (seats 60 to 68).

#### 4.2.2 Activity hotspots

**Fig 17:**
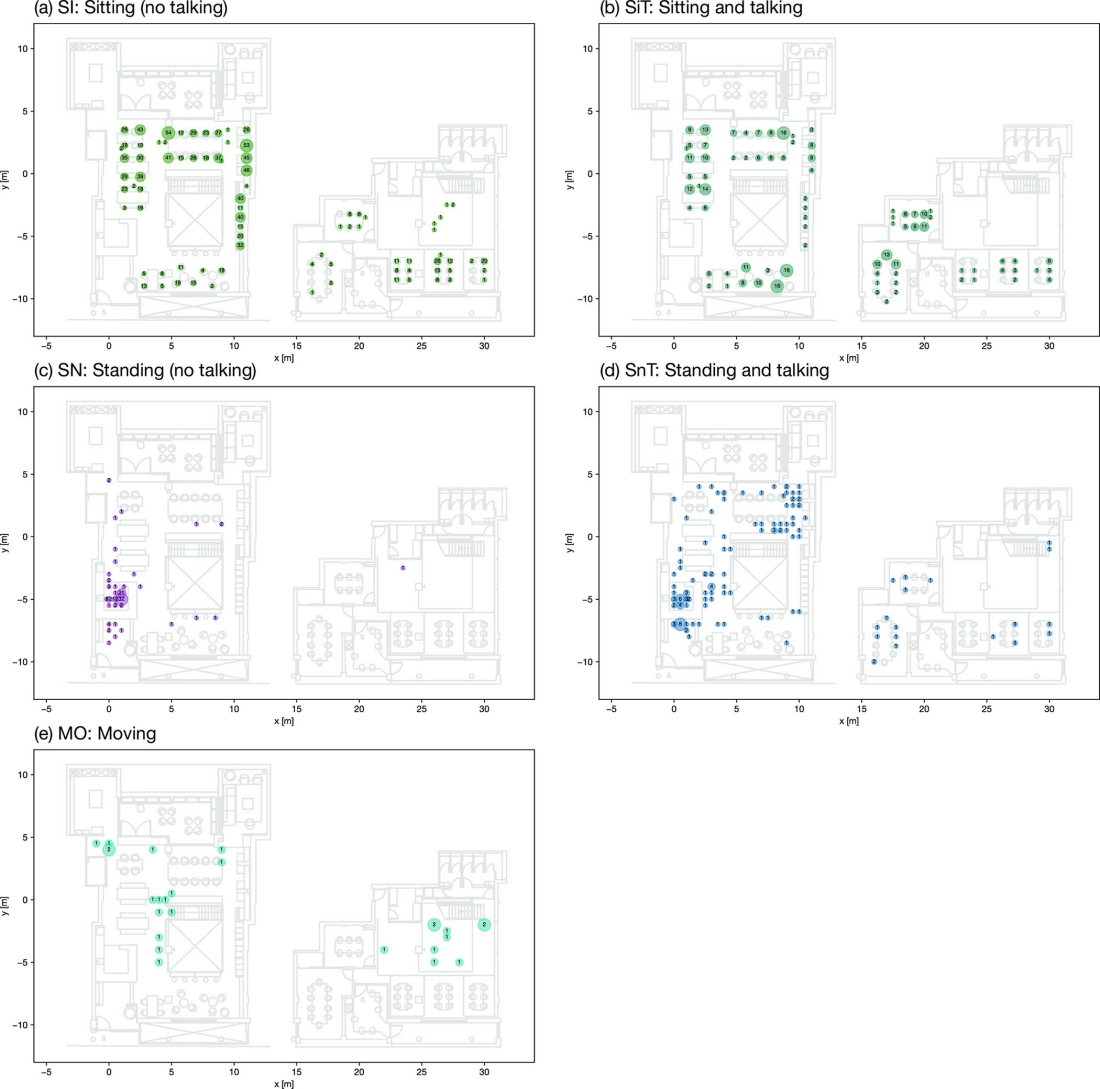
Aggregation of activity observations.

*Hotspots by aggregation*. The popular areas are first identified by aggregating the count of observations in the five activity categories SI, SiT, SN, SnT and MO, as demonstrated by Fig 17. The most popular seats for sitting without talking (SI) are seat no.0, 11 and 13, while the most popular seats for sitting and talking (SiT) are no.4, 22 and 23. Also, a relatively large number of occupants sitting and talking in meeting rooms are identified. The major standing activities are found around the reception area, while people tend to stand and talk at the circulation area near the staircase on the ground floor as well. Most of the movement activities are observed around the central staircase.

**Fig 18:**
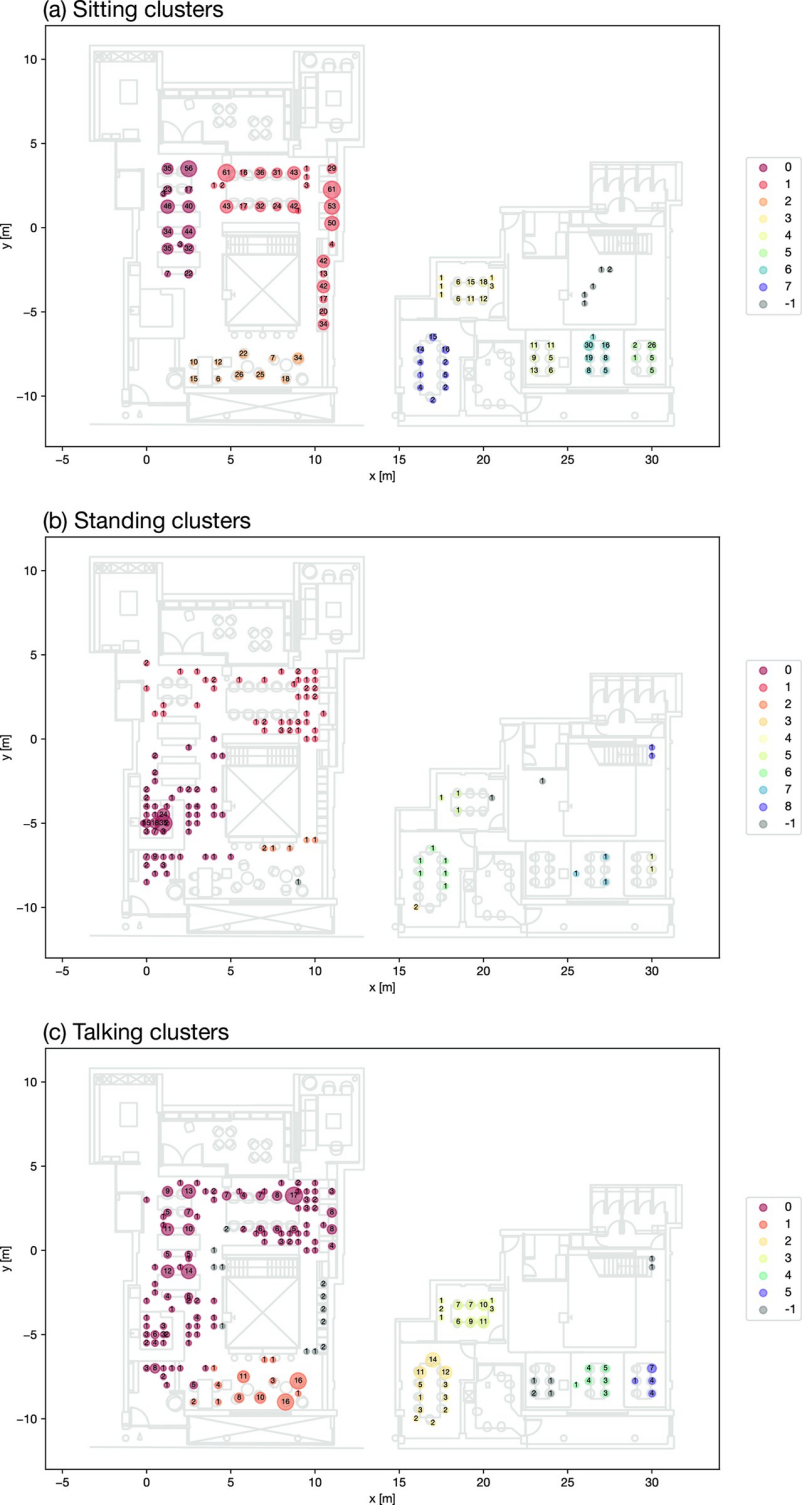
DBSCAN cluster results for activities.

*DBSCAN cluster analysis of activity data*. DBSCAN cluster analysis is applied to three restructured datasets: Sitting (SI and SiT), Standing (SN and SnT) and talking (SiT and SnT), with the results shown in Fig 18(A)–18(C) respectively. Different clusters are represented by various colours, with the count on each coordinate noted. The parameters eps and min_samples applied in the clustering and the number of clusters, number of noise points and silhouette score in results are shown in Table 5. The silhouette values are at a good quality with the scores of 0.52, 0.66 and 0.45 separately.

**Table 5:** Activity clustering parameters.

	No of clusters	Noise points	Silhouette score	Eps	Min_samples
Sitting clusters	8	6	0.516	1.5	15
Standing clusters	9	3	0.656	2	2
Talking clusters	6	25	0.449	1.5	15

For sitting activities, the coordinates are clearly clustered based on the layout of seating areas, with eight clusters and a noise cluster identified. Three largest clusters 0,1 and 2 are found on the ground floor. This suggests that the majority of sitting activities for work purposes are concentrated on the ground floor, with relatively fewer observed in the basement area. The coordinates for standing activities form nine clusters along with a noise cluster. Most of these clusters also concentrate around the ground floor, with two major concentration areas: one near the reception and the other in the circulation space at the top right corner. The talking activities, indicating where the communications take place around the space, are clustered to six groups with one noise group. It suggests that the talking and interacting activities are found around the large tables that can accommodate four to ten people, and the sofa and round tables near the entrance are also popular for sit-down communications. In the basement, two enclosed meeting rooms are the major spaces where communications occur, as expected. Occupants also add seats or move to seats around in the meeting room.

## 5 Discussion

In the context of hybrid working, it is especially important to improve the flexibility and diversity in an office space and fit the changing pattern for space utilisation. The occupancy information could contribute to better space planning and effective reconfiguration [13]. Although a range of studies have attempted to capture the occupancy and activities, a very limited number of studies discuss how to inform the design and utilisation strategies, especially in the co-working space setup. This study attempts to fill this gap with some exploratory insights.

The findings of this study support the notion that occupants exhibit clear preferences for working time and specific types of seats based on their needs and preferences in a hybrid working setting, despite the availability of a diverse and flexible range of seating options. It is evident that occupants may prefer certain areas for different work tasks, as some seats are more utilised than others, while some features from the popular seats could be generalised. This highlights the importance of understanding and catering to these preferences in co-working office design to enhance the flexibility of the space and well-being of occupants.

More specifically, the variation in occupancy levels over time in the co-working space aligns with the new hybrid work patterns identified in post-pandemic surveys [1, 67]. The increase in occupancy levels from July 2021 coincides with the easing of COVID-19 restrictions in England during the spring and summer [68], indicating employees’ eagerness to return to the office after the lockdown. The stabilization of occupancy levels in more recent times suggests a shift towards adapting to hybrid working, with an observed weekly seasonality pattern [66]. The employees still tend to stick to normal working hours from 10am to 5pm. Tuesday, Wednesday and Thursday have higher occupancy rates, while occupants may be more inclined to stay at home for Monday and Friday. This indicates that employees tend to prioritize in-person work during the middle of the week. Additionally, afternoon time, particularly from 2 pm to 4 pm, appears to be a popular time for meetings as indicated by the higher occupancy of enclosed meeting rooms during that period. The annual pattern suggests that autumn is a season with relatively higher occupancy levels, indicating a preference for in-person work during that time. The variation in December may be attributed to the Christmas event and the associated long holiday. This information has significant potential for informing future workspace planning, providing evidence on estimating occupancy and optimizing space utilization. It suggests that a maximum capacity of 30 to 45 occupants is suitable for a normal working day, allowing for appropriate space planning. The identification of specific time periods, such as the popular afternoon meeting times, can guide scheduling and availability of meeting rooms to meet occupants’ needs. However, it is important to carefully determine the time granularity level for calculating seat utilization rates, as different granularities can lead to significant differences in the results [49]. It may require further investigations to determine the suitable way of calculating seat utilisation rates.

**Fig 19:**
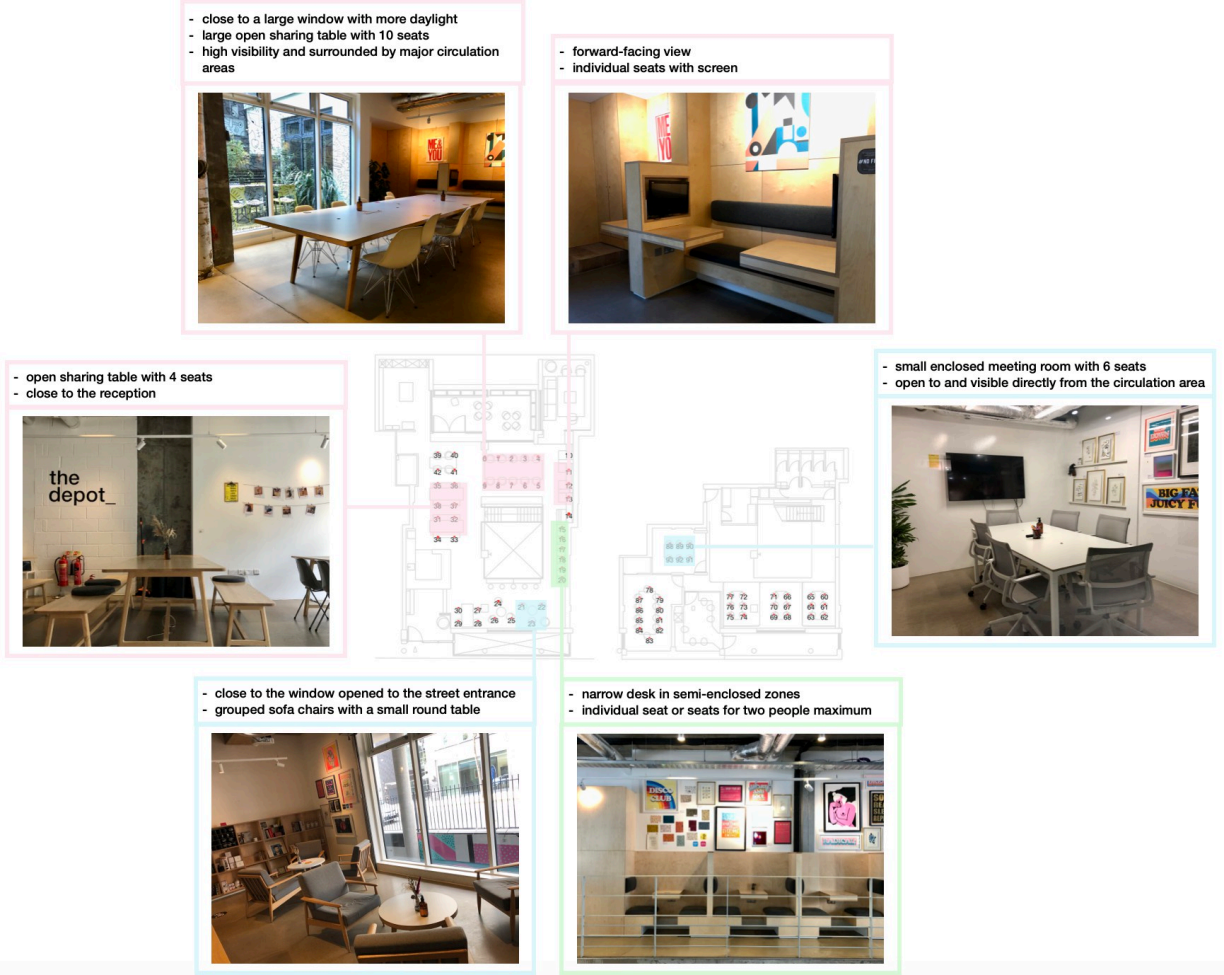
Identified seating hotspots.

The preferences of occupants for different zones and types of seats in a hot-desking system are revealed through the analysis of occupancy and activity hotspots using techniques like KDE and DBSCAN cluster analysis. The identification of occupancy and activity hotspots supports the interpretation of which type of seat or what design elements may be more attractive to the users; subsequently, it provides evidence for the future design. Fig 19 illustrates the popular seat types identified through spatial hotspot analysis. The pink zone represents spaces suitable for both talking and working, the blue zone indicates areas where more communication takes place, and the green zone represents the area for focused work. Some characteristics of the seats are included in the figure. The seating areas with higher visibility and openness seem to be preferable for communication, while the semi-enclosed spaces are more popular for focused working. These findings align with previous studies that have shown the preference for seats next to windows due to their positive impact on productivity, health, and well-being [69, 70]. The table near the entrance (seat 28 to 30) and the table immediately next to the reception (seat 33 and 34) are the least occupied areas on the ground floor, indicating a potential preference for staying in a bit of distance away from the reception area. Furthermore, the open meeting spaces in the basement, designed to accommodate both meetings and focused work, are less utilized. The reception area plays a key role in triggering casual interactions, particularly at the beginning and end of the day, and corridor chats near major workstations are frequently observed [71].

## 6 Conclusion

In the new future of work, enhancing the flexibility in work has a significant implication on improving employees’ well-being and work satisfaction. The workspace plays an important role in providing a flexible and diverse environment to encourage workers to come to office and work together. Co-working space is expected to accommodate the rising desire for flexibility and inclusivity in work environment for hybrid workers with its unique feature as a locally accessible working hub.

Analysing the occupancy and activities around the workspace enhances the understanding of the use of space, especially in the hybrid and flexible working setup. The identification of trends and patterns subsequently contribute to space optimisation, user satisfaction, function and resource allocation, operational efficiency and future planning. Also, the temporal occupancy count variation has a potential to inform the energy calculation and simulation as a schedule for the space [72]. By leveraging insights from occupancy patterns and activity hotspots, co-working spaces can create environments that meet the diverse needs of occupants, supporting their productivity, collaboration and overall well-being.

Our data-driven study fills the knowledge gap by demonstrating the use of sensor-captured occupancy data and onsite-observed human activity data in informing space planning and design. It integrates the space utilisation concepts and spatiotemporal data analysis uniquely with two types of data. In this case, the observed human activities act as a useful supplement to the sensor-detected occupancy to understand the space preference of occupants for different activities. The analysis identifies the variation of occupancy over different time period and the spatial hotspots for occupancy and activities. It first captures and validates the changes in work pattern after the pandemic, showing the willingness of workers to come back to office. Occupants tend to adhere to a traditional 10am to 5pm routine, predominantly on Tuesday, Wednesday and Thursday. The findings also help discover the types of seating area preferred by occupants. For example, we found that certain seating areas, such as those positioned near windows or in more open, connected and visible locations like large tables, were consistently favoured for communication and collaborative activities. These areas seemed to facilitate social interaction, teamwork, and spontaneous discussions among occupants. On the other hand, clustering results reveal that individuals seeking a quieter and more focused work environment tended to gravitate towards semi-enclosed or secluded seating options. These spaces provided a sense of privacy and concentration, allowing individuals to engage in deep work or tasks requiring high levels of concentration without distractions. Overall, the findings highlight the interplay between seat preference and human behaviour within the co-workspace. We show that, by understanding these dynamics, designers and workspace managers can tailor the environment to accommodate and enhance different work styles and preferences. It contributes to the overall objectives of accommodating diversity and making the post-pandemic workspace more inclusive.

The study has certain limitations. Firstly, the sensor-detected occupancy data may be subject to errors and inaccuracies, and it may not capture certain factors such as the presence of pets or minor seat alterations. The sensor-detected occupancy, especially on seats, can vary from the actual occupancy. Sensors are unable to identify short-stay guests around the space. The size of the dataset also presents challenges in performing complex distance calculations. Moreover, although the occupants’ awareness of observation and sensors are minimised in the experiment protocol, the reactivity bias may be inevitable. Secondly, the collection of activity observation data was relatively small and may not fully capture the range of activity variations in the space, while the suggested observation time is about two to three weeks [73]. Also, it is hard to capture the exact coordinates through the observation, which is not as precise as the sensor-detected data. Furthermore, one persisting and inevitable major challenge in occupancy analysis is the inherently unpredictable nature of occupants [32]; therefore, this study only aims to find out the spatial pattern identified from a collection of people without specifying the unique individual cases. The study is based on a specific co-working space and the ability to generalize has not been tested. Further research is needed to validate and expand upon these findings, taking into account a larger sample of co-working spaces and considering additional factors such as organizational culture and work practices.

Moving forward, future research should aim to address these limitations, and further explore how to improve flexibility and well-being through the design of space. This opens a new line of enquiry using big data for evidence-based workspace design. Decoupling the spatial association between environmental factors (such as thermal comfort, daylight, view to window, and ventilation) and occupancy patterns can provide deeper insights into the reasons why certain spaces are preferred by the occupants and subsequently suggest for the design alternation for better space utilisation. Additionally, supervised machine learning approaches can be developed to forecast space utilization using environmental datasets which is essential for data-driven human-centric design.
